# Thermophysical Property Measurements of Tetrabutylphosphonium Oxalate (TBPOx) Ionic Semiclathrate Hydrate as a Media for the Thermal Energy Storage System

**DOI:** 10.3389/fchem.2020.00547

**Published:** 2020-07-16

**Authors:** Takashi Miyamoto, Ryo Koyama, Naruki Kurokawa, Atsushi Hotta, Saman Alavi, Ryo Ohmura

**Affiliations:** ^1^Department of Mechanical Engineering, Keio University, Tokyo, Japan; ^2^Department of Chemistry and Biomolecular Sciences, Ottawa University, Ottawa, ON, Canada

**Keywords:** ionic semiclathrate hydrate, tetrabutylphosphonium oxalate (TBPOx), carboxylic acid, phase equilibrium temperature, dissociation heat, phase change material (PCM), thermal energy storage medium

## Abstract

With increasing global power demand, thermal energy storage technology could play a role ensuring a sustainable energy supply in power generation from renewable energy sources and power demand concentration. Hydrates have high potential as phase change materials (PCMs) for the use as a thermal energy storage medium. To develop thermal energy storage technology using a hydrate-based material, further investigation of thermophysical properties and the selection of a suitable hydrate are required. Tetrabutylphosphonium oxalate (TBPOx) ionic semiclathrate hydrate contains oxalic acid in salt form, as a guest compound, which is classified as carboxylic acid group with low environmental impact. In the present study, the phase equilibrium temperature and the dissociation heat of TBPOx hydrate were measured. The highest equilibrium temperature of the solid hydrate formed was 9.4°C at the mass fraction 0.35 of TBPOx in aqueous solution. The largest dissociation heat was 186.0 ± 0.5 kJ·kg^−1^ at the mass fraction of 0.35. Comparing with other PCMs with close phase equilibrium temperatures, TBPOx hydrate is superior in safety and sustainability. These results indicate that TBPOx hydrate would be suitable as the thermal storage medium for the general air conditioning systems.

## Introduction

To deal with the world energy consumption issue, modern society needs energy management to relieve the concentration of electricity demand, which leads to emission of greenhouse gas from power generation (International Energy Agency IEA World Energy Outlook, 2019). The amount of generated electricity throughout the day depends on the time of day at a particular location. The energy use in the daytime tends to be higher than the night time. Additionally, renewable energy technology utilizing sources such as solar and wind power depends on the operating environment. These energy sources are unfavorable to achieve a stable electricity supply at all times. Current energy supply system management requires solutions to close the huge gap between the production and consumption of the energy, particularly when the contributions from renewable energy are included.

Thermal energy storage systems could help to change these imbalances between energy supply and demand (Pielichowska and Pielichowski, 2014; Veerakumar and Sreekumar, 2015; Pelay et al., 2017). The thermal energy storage is the technology that stores heat energy using the heat capacity of substances. At night, the surplus electricity would be stored as the cold energy in advance of the daytime peak in power usage. When daytime power demand increases, by using the cold thermal energy stored in the substances, it could help to reduce the total electricity that needs to be generated. These systems use phase change materials (PCMs) as the thermal energy storage medium. These substances can utilize latent heat adding to sensible heat and have higher energy density than sensible heat storage (Mehling and Cabeza, 2007, 2008).

The main categories of PCMs are organic and inorganic compounds in solid–liquid PCMs (Pielichowska and Pielichowski, 2014; Veerakumar and Sreekumar, 2015). The organic PCMs are represented by paraffin, and the typical examples of the inorganic PCMs are metal salt hydrates and water. Having flammability and low thermal conductivity, organic PCMs have safety issues (Zalba et al., 2003). Metal salt hydrates have corrosiveness and need high temperatures to melt (Veerakumar and Sreekumar, 2015). With those characteristics, metal salt hydrates are not suitable for long-term use. Water is often used as ice. Despite having the large latent heat, the freezing point of ice is relatively low. Therefore, the available temperature range of ice is limited. Instead of these compounds, recent studies proposed clathrate hydrates or more simply hydrates as thermal energy storage medium (Li et al., 2012; Castellani et al., 2014; Oshima et al., 2018).

Clathrate hydrates are crystalline compounds consisting of the space-filling cages of water molecules and other molecules called guest compounds enclosed within the cages. They show various physical properties depending on the type of guest compounds contained (Nakayama and Hashimoto, 1980; Nakayama et al., 1983; Mayoufi et al., 2011; Sakamoto et al., 2011; Sato et al., 2013; Yamauchi et al., 2017a,b; Arai et al., 2018; Shimada et al., 2018, 2019). Ionic semiclathrate hydrates have unique structures. The anion of guest molecules replaces water molecules of the cage and the cation of guests is enclosed within the hydrogen-bonded water molecules cages. In the process of synthesizing ionic semiclathrate hydrates, tetrabutylammonium (TBA) salts or tetrabutylphosphonium (TBP) salts have mainly been found convenient for use. With the hydrogen bonding between water within the cage molecules, and the strong interaction between the guest and water molecules, these substances have large dissociation heats (Alavi and Ohmura, 2016). The decomposition phase transformation of ionic semiclathrate hydrates can occur under atmospheric pressure and around the room temperature. Consisting of water molecules, they are flame-retardant compounds. Based on these beneficial properties, the ionic semiclathrate hydrates can be used as PCM.

In recent years, tetrabutylammonium bromide (TBAB) hydrate has been commercialized as PCM for the air conditioning refrigerant (Darbouret et al., 2005; Wang and Dennis, 2015). It is the only commercialized semiclathrate hydrate and includes a bromide halogen ion within the guest compounds. The previous studies reported the equilibrium temperature of TBAB hydrate as 12.7°C and the largest dissociation heat as 193.2 kJ·kg^−1^ (Oyama et al., 2005; Kobori et al., 2015). Data of thermophysical property are essential to select suitable materials as thermal energy storage media. For the development of thermal energy storage technology, similar experiments on other hydrates should be conducted.

The semiclathrate hydrates composed with anions with carboxylic acid anions in the guest compounds have been previously investigated (Nakayama and Torigata, 1984; Yamauchi et al., 2017a,b; Arai et al., 2018; Shimada et al., 2018, 2019). Carboxylic acid anion containing compounds are more environmentally benign, unlike those which incorporate halide anions. The previous studies of ionic semiclathrate hydrates with carboxylic acid anions imply the existence of relationships between thermophysical properties of ionic semiclathrate hydrates and the molar mass of the guest compounds. From the trend of physical property values, the heats of dissociations are expected to be large when the range of molar mass of the carboxylic acid in the guest compounds is from 60 to 90 kg·kmol^−1^.

Tetrabutylphosphonium oxalate (TBPOx) hydrate is an ionic semiclathrate hydrate consisting oxalic acid as the guest compound in oxalate anion form. The molar mass of oxalic acid is 90.03 kg·kmol^−1^, and this corresponds to the molar mass range of the carboxylic acid of the guest group in semiclathrate hydrates that are expected to have large dissociation heat. A previous study reported on the synthesis and properties of the ionic semiclathrate hydrate with the oxalic acid anion, namely, tetrabutylammonium oxalate (TBAOx) hydrate (Dyadin et al., 1976). The highest phase equilibrium temperature of TBAOx hydrate is reported as 16.2°C. However, no data about the other thermophysical properties were measured. The phase equilibrium temperatures of TBP hydrates have the tendency to be lower than those of TBA hydrates when both hydrates have the same guest anions (Kobori et al., 2015). By using TBP salt instead of TBA salt, the phase equilibrium temperature of TBPOx hydrate could be expected to be lower than TBAOx and it would meet the required temperature of the cooling medium for the air conditioning systems (ASRAE, 2016).

TBP hydrates consisting of carboxylic acid anions were reported in the previous studies (Arai et al., 2018; Shimada et al., 2018, 2019). Further investigation of the thermophysical properties of hydrate will lead to expand the application range of hydrates. Depending on the obtained properties, it could be applied to the various hydrate technologies. In this study, phase equilibrium temperatures and dissociation heats of TBPOx hydrate were measured.

## Materials and Methods

### Materials

The materials used in this study are summarized in Table 1. TBPOx aqueous solutions were obtained by neutralizing tetrabutylphosphonium hydroxide (TBPOH) aqueous solutions (0.40 mass fraction in aqueous solution, Sigma-Aldrich Co., LLC.) with oxalic acid (0.98 content in anhydrous solid, FUJIFILM Wako Pure Chemical Co., Ltd.). Before the neutralization, oxalic acid solid and distilled water in the vessels were warmed with hot water until oxalic acid solid was completely dissolved in the solution. The distilled water for the concentration adjustment was made by a water distillation unit (Yamato Scientific Co., Ltd., WG 222) in the laboratory. In this study, the samples of TBPOx aqueous solution were made at the mass fraction range from 0.20 to 0.40. The mass of oxalic acid, water, and TBPOx aqueous solution was measured by an electronic balance (IUW-200D sefi, As One Co., LLC.) with an expanded uncertainty of ±0.1 mg (coverage factor *k* = 2).

**Table 1:** Specifications of materials used in this study.

**Name**	**Chemical formula**^**b**^	**Supplier**	**Purity**
Oxalic acid	H_2_C_2_O_4_	Sigma-Aldrich Co. LLC.	0.98 content in anhydrous solid
Tetrabutylphosphonium hydroxide (TBPOH) aqueous solutions	(CH_3_CH_2_CH_2_CH_2_)_4_POH	Sigma-Aldrich Co. LLC.	0.40 mass fraction in aqueous solution^a^
Water	H_2_O	Laboratory made	Electrical conductivity was <0.1 μS·cm^−1^
Tetrabutylphosphonium oxalate (TBPOx) aqueous solutions	[(CH_3_CH_2_CH_2_CH_2_)_4_P]_2_C_2_O_4_	Laboratory made with above materials	The standard uncertainty of mass fraction was ± 5.0 × 10^−3^ ^b^
Hydrochloric acid	HCl	Kanto chemical Co. Inc.	0.01 mol·L^−1^ ^c^

a *This is the labeled mass fraction on the reagent bottle. The concentration with the uncertainty of TBPOH aqueous solution was measured by acid-based titration with HCl. The actual mass fraction of TBPOH aqueous solution was 0.41. The standard uncertainty of the mass fraction was 5.0 × 10^−3^*.

b *The uncertainties of the mass fraction of TBPOx aqueous solution were estimated from the uncertainties of the mass measurements on TBPOH aqueous solution, oxalic acid solution, and water on the neutralizing and adjusting processes*.

c *HCl was utilized for the titration measurement to determine the uncertainty for mass fraction of TBPOH aqueous solution*.

### Equilibrium Temperature Measurement

**Figure 1:**
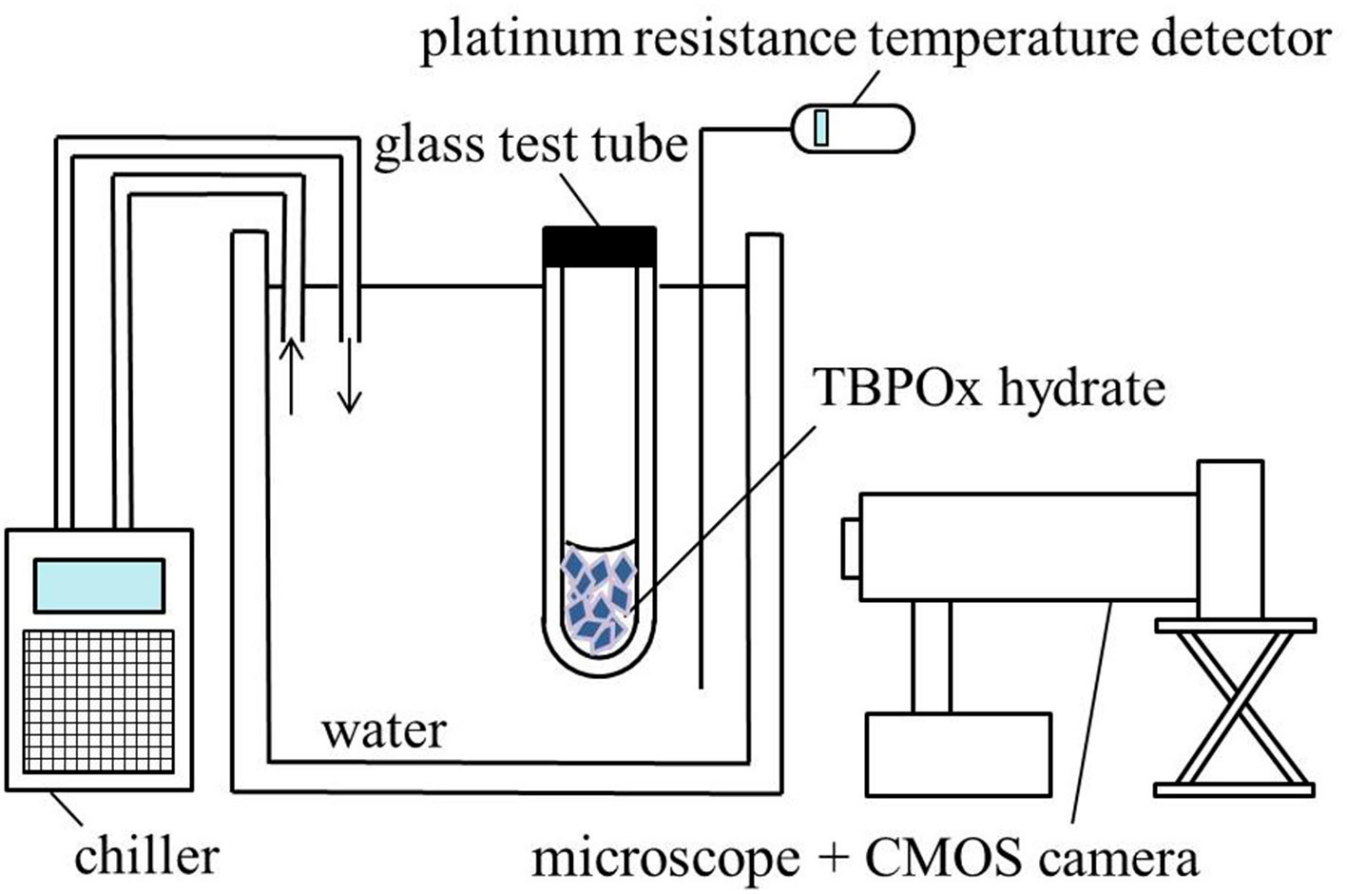
Schematic diagram of the equipment for the measurement of the phase equilibrium temperature.

The schematic diagram of experimental equipment used for observing the dissociation of TBPOx hydrate crystal is shown on Figure 1. Approximately 0.5 g of TBPOx aqueous solution samples in the glass test tubes was refrigerated at −20°C for 24 h to form the hydrate crystals. After visually confirming the hydrate formation, the glass test tubes (external diameter 10 mm, internal diameter 8 mm, height 90 mm) within the hydrate crystal were set into the water bath under the atmospheric pressure. The temperature of the water was measured by a platinum resistance temperature detector with an expanded uncertainty of ±0.1°C (coverage factor *k* = 2) and controlled by a chiller (Tokyo Rikakikai Co., CTP-3000). The system temperature was maintained constant for at least 4 h. The crystal dissociation behavior was visually observed by a CMOS camera with a microscope. If no remarkable change was seen on the crystals, the system temperature was increased by 0.1°C and maintained constant another several hours again. When the dissociation was observed, the system was kept at the same temperature for several hours. By repeating these procedures, the hydrate crystals were completely dissociated. The equilibrium temperature was the temperature just before complete dissociation. Therefore, the equilibrium temperature was determined as 0.1°C lower than the dissociation temperature.

The measurements were performed at least three times at each mass fraction. Three different samples prepared from three different TBPOx aqueous solutions were measured for a given mass fraction. The visual observations with the similar apparatus and method were performed in the previous studies (Yamauchi et al., 2017a,b; Arai et al., 2018, 2019; Shimada et al., 2018; Koyama et al., 2019). These studies supported the reliability of the equilibrium temperature obtained in this study.

### Dissociation Heat Measurement

The dissociation heats of TBPOx hydrate were measured by the differential scanning calorimetry (abbreviated as DSC) (TA Instrument, DSC25). For the temperature and latent heat calibration, indium (purity 99.99%) and distilled water were used. The uncertainty of enthalpy was confirmed by measuring the latent heat of water three times. The average of three measurements was 335.7 kJ·kg^−1^ and the uncertainty of the dissociation heat measurements was estimated to be 3.2 kJ·kg^−1^ (coverage factor *k* = 2), which was consistent with the literature data within the experimental uncertainty (Feistel and Wagner, 2006).

Approximately 15 mg of sample from TBPOx aqueous solution and air was encapsulated into the aluminum test cell as a sample and a reference. The test cells have a volume of 40 × 10^−3^ cm^3^. The sample cell and the test cell were placed inside the DSC device filled with dry N_2_ gases at 0.1 MPa to eliminate moisture.

**Figure 2:**
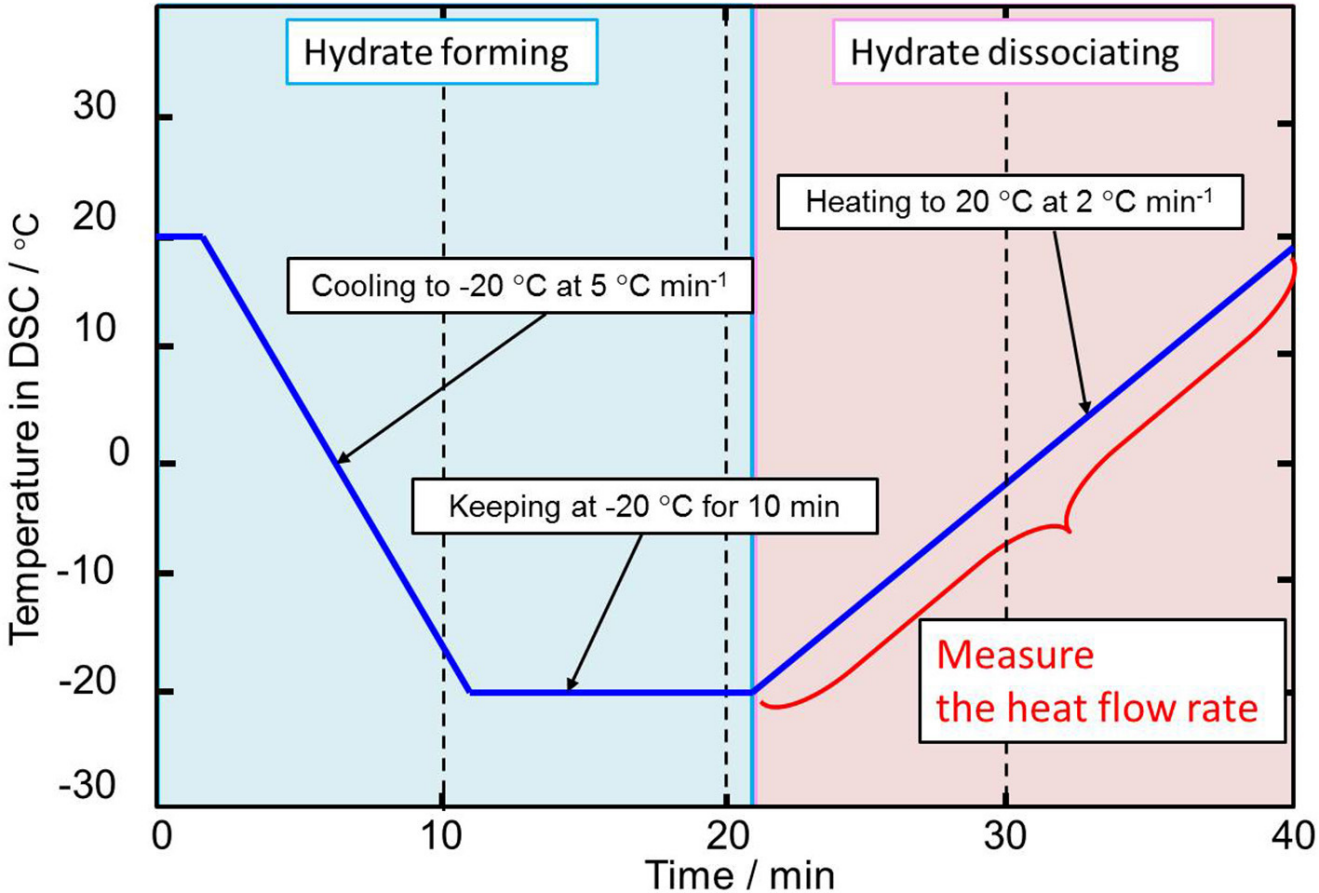
Schematic diagram of the DSC temperature program for the dissociation heat measurement.

The temperature in the DSC was controlled as shown in Figure 2. First, the temperature was kept for 20°C to dry the internal device, and then it was decreased from 20 to −20°C under a cooling rate of −5°C min^−1^ to form the hydrate crystals. After keeping at −20°C for 10 min to complete the formation of the hydrate crystals, the system temperature was increased from −20°C to 20°C under a heating rate of 2°C min^−1^ to dissociate the hydrate crystals. The reliability of the higher heating rate of 2°C min^−1^ used in this process in determining the dissociation temperature was shown in previous studies (Yamauchi et al., 2017a; Arai et al., 2018; Shimada et al., 2018).

During the hydrate dissociating process, the heat flow rate, which was normalized per unit mass, was measured. Then, the dissociation heat of TBPOx hydrate was obtained by integrating the endothermic peak of the heat flow rate. As the equilibrium temperature measurement, triplicate measurements were performed at each mass fraction of TBPOx solutions. Three independent measurements were performed with three different samples for a given mass fraction.

## Results and Discussion

### Phase Equilibrium Temperatures

**Figure 3:**
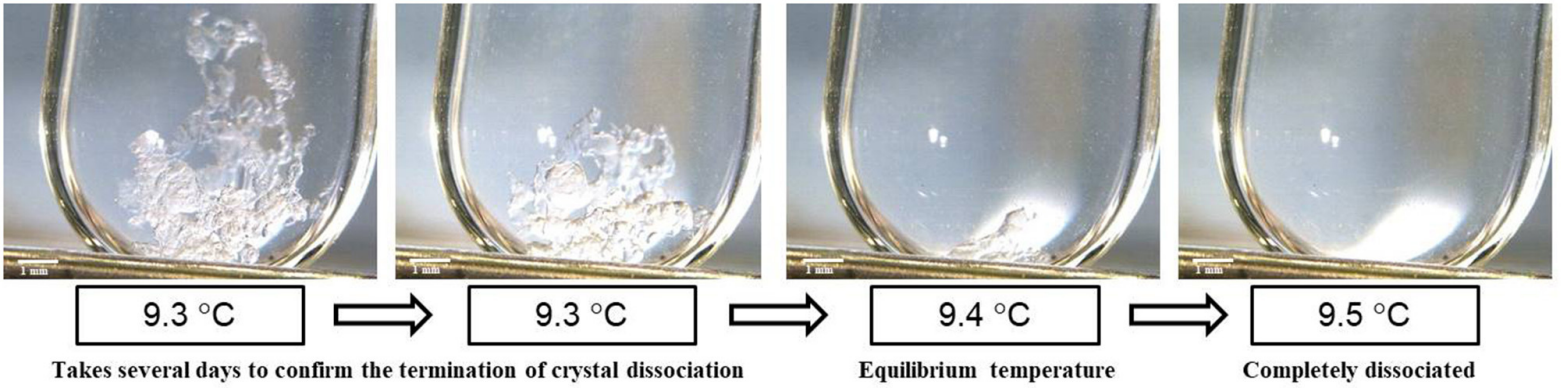
The visual observation during TBPOx hydrate dissociation.

Phase equilibrium temperatures of TBPOx hydrate were measured at the mass fraction range from 0.20 to 0.40 under the atmospheric pressure by visual observation. The visual observation images of hydrate dissociation at a mass fraction of 0.35 are shown in Figure 3. This figure also reveals the experimental process to measure the equilibrium temperature of TBPOx hydrate. Near the equilibrium temperature, it took several days to confirm the termination of hydrate dissociation with one step of increasing temperature. Constant temperature increasing methods, like DCS measurement, would not be suitable for the observation of the slow hydrate dissociation dynamics. These results obtained from the process similar to Figure 3 are presented in Table 2, where *w*_TBPOx_, *x*_TBPOx_, and *T*_eq_, respectively, indicate the mass fraction, the mole fraction of TBPOx aqueous solution, and the phase equilibrium temperatures.

**Table 2:** The phase equilibrium temperatures of TBPOx hydrate.

***w*_**TBPOx**_^**a**^**	***x*_**TBPOx**_^**b**^**	***T*_**eq**_^**c**^ (^**°**^C)**
0.20	0.0074	6.2
0.25	0.0098	7.6
0.30	0.0126	8.6
0.34	0.0151	9.3
0.35	0.0157	9.4
0.36	0.0164	9.3
0.37	0.0171	9.2
0.38	0.0180	9.2
0.39	0.0186	9.3
0.40	0.0194	8.0

a *The standard uncertainty u (w_TBPOx_) is ± 5.0 × 10^−3^*.

b *The standard uncertainty u (w_TBPOx_) is ± 4.0 × 10^−4^. x_TBPOx_ indicates the mole fraction of TBPOx in aqueous solutions. These values of mole fractions were obtained by converting the mass fractions*.

c *The expanded uncertainty U (T_eq_) is ± 0.1 K (k = 2)*.

In the measurement, the maximum points were confirmed at a mass fraction of 0.35. The phase equilibrium temperatures significantly increased with the mass fraction at a mass fraction range below 0.34. In the ranges from 0.34 to 0.35, the phase equilibrium temperature showed a slight increase. Then, the phase equilibrium temperatures gradually decreased with the mass fraction increase in the range from 0.35 to 0.39. For the solutions over the mass fraction range 0.39, the phase equilibrium temperatures sharply decreased.

In the previous studies of ionic semiclathrate hydrates, similar tendencies of temperature increase and decrease with mass fraction in solution were reported (Yamauchi et al., 2017a,b; Arai et al., 2018, 2019; Shimada et al., 2018, 2019). From the low mass fraction to a specific mass fraction for each ionic semiclathrate hydrate, the phase equilibrium temperature tended to rise with the increase of the mass fraction. After a specific mass fraction, the phase equilibrium temperature decreased with the increase of the mass fraction in solution. At that specific mass fraction, the highest phase equilibrium temperature was observed. The previous studies also reported that the congruent point would be at the mass fraction of the highest phase equilibrium temperature (Yamauchi et al., 2017a,b; Arai et al., 2018, 2019; Shimada et al., 2018, 2019; Koyama et al., 2019).

In the present study, the congruent point would exist at the range of mass fraction 0.34 to 0.36. The highest phase equilibrium temperature was 9.4°C at the mass fraction 0.35, which met the required temperature of the cooling medium for the air conditioning system from 5 to 15°C (ASRAE, 2016).

### Heat Flow and Dissociation Heat

**Figure 4:**
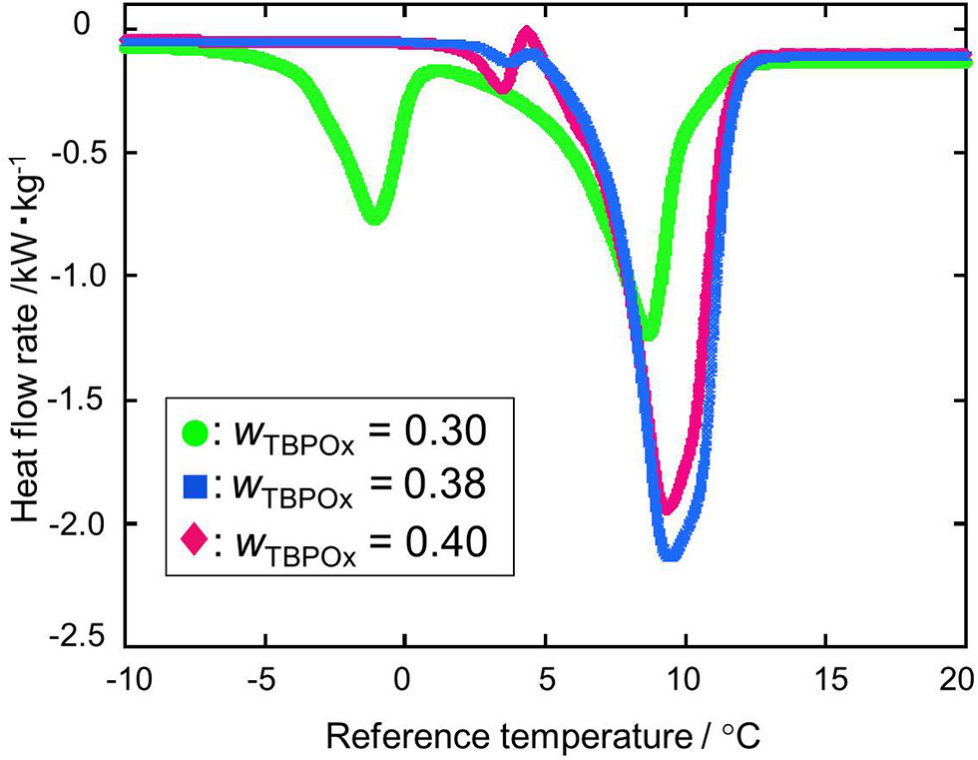
The heat flow rate at the mass fractions 0.30, 0.38, and 0.40: green •, *w*_TBPOx_ = 0.30; blue ■, *w*_TBPOx_ = 0.38; red ♦, *w*_TBPOx_ = 0.40.

**Figure 5:**
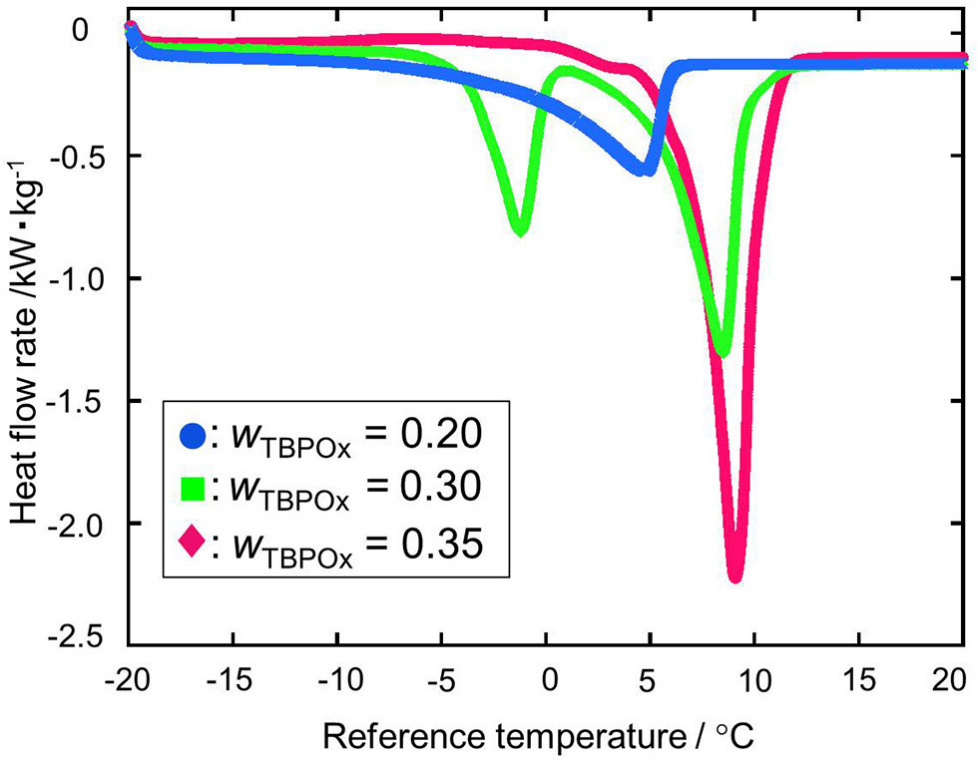
The heat flow rate at the mass fractions 0.20, 0.30, and 0.35: blue •, *w*_TBPOx_ = 0.20; green ■, *w*_TBPOx_ = 0.30; red ♦, *w*_TBPOx_ = 0.35.

**Figure 6:**
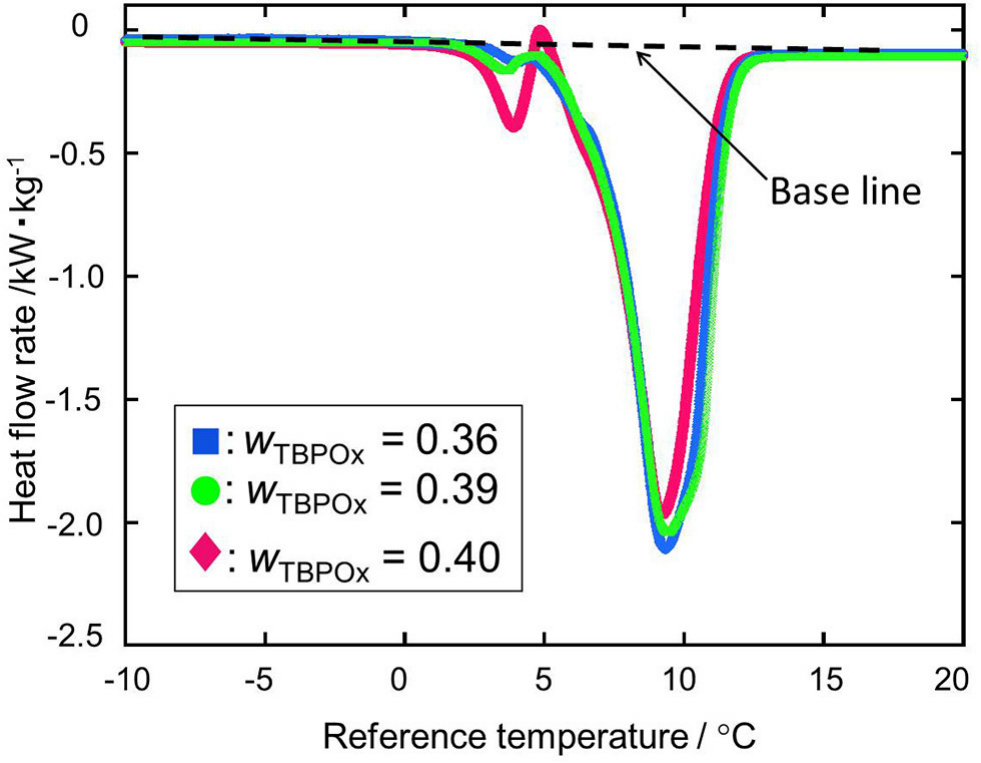
The heat flow rate at the mass fractions 0.36, 0.39, and 0.40: blue ■, *w*_TBPOx_ = 0.36; green •, *w*_TBPOx_ = 0.39; red ♦, *w*_TBPOx_ = 0.40.

**Figure 7:**
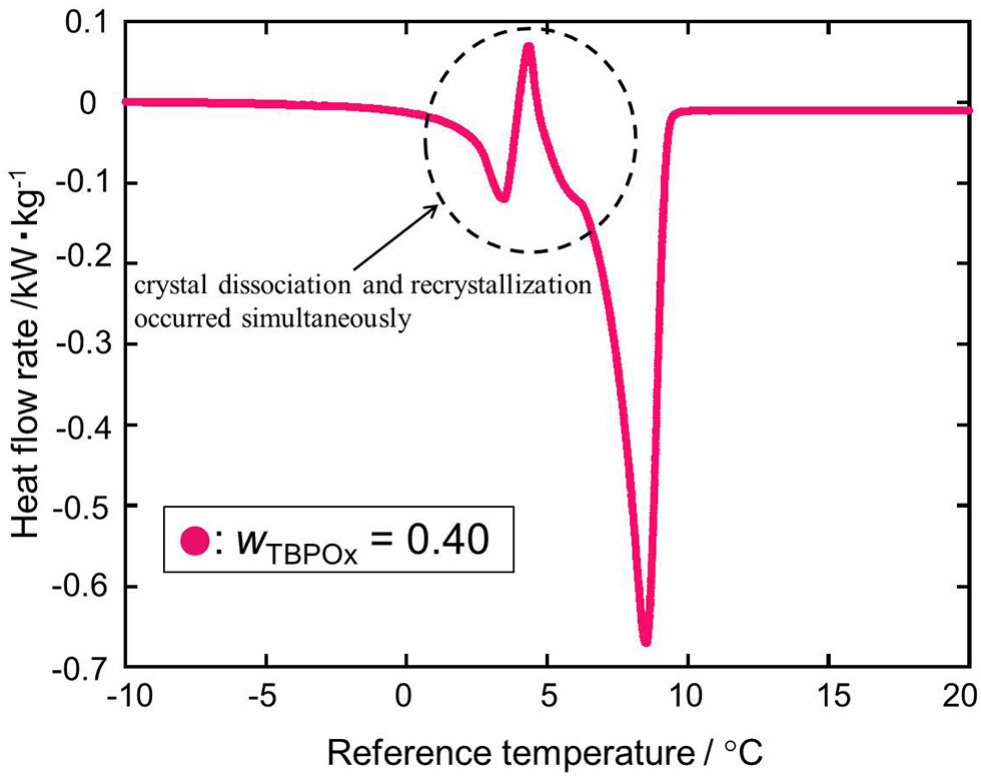
The heat flow rate at the mass fractions 0.40 with the heating rate of 0.5°C/min: red •, *w*_TBPOx_ = 0.40.

The dissociation heats of TBPOx hydrate were deduced from the data of the heat flow rates. In obtaining the dissociation heats, the baseline was drawn from the start to the end of the heat flow peak to integrate the heat flow rates. TBPOx solutions were injected into the aluminum pan, which is the test section. In the present measurements, the thermal behavior of TBPOx hydrates formed inside the pans was observed. The heat flow rates were measured by DSC at nine different mass fractions from 0.20 to 0.40. The results are presented in Figures 4–**7**. As shown in Figure 4, three representative heat flows were respectively obtained in the measurement range at each mass fraction 0.30, 0.38, and 0.40.

The first peak of the mass fraction 0.30 at a temperature around 0°C emerged due to ice melting, which was observed at low mass fraction in the measurement range. The ice would be formed by residual water that was not consumed for hydrate crystal formation. The largest peak of the mass fraction 0.30, 0.38, and 0.40 at a temperature around 10°C was due to hydrate dissociation, which was obtained at almost all mass fractions in the present measurements. Considering the starting point of the heat flow, the small peak at the temperature around 5°C was not caused by the ice melting. This temperature indicated that the small peak was due to the dissociation of a hydrate phase, which has a different equilibrium temperature from the hydrate dissociated around 10°C. Polymorphism was observed in the previous studies of ionic semiclathrate hydrates (Oyama et al., 2005; Sakamoto et al., 2011; Yamauchi et al., 2017a,b; Arai et al., 2018, 2019). In the TBPOx hydrate system, at least two types of hydrates with different thermophysical properties and crystal structures would exist at a mass fraction of 0.40.

Figure 5 shows the heat flows at the mass fraction range from 0.20 to 0.35. The heat flow curves significantly changed with the increase in mass fraction. At the mass fraction 0.20, the endothermic peak due to ice melting did not appear around 0°C. The peak appearing at the midpoint between 0 and 10°C was due to only hydrate dissociation. Since the nucleation of ice is a stochastic phenomenon, no ice formation from the residual water occurred at a solution mass fraction of 0.20. From the mass fraction 0.30 to 0.35, the hydrate dissociation peak increased while the ice melting peak decreased. The deepest peak was observed at the mass fraction 0.35.

Figure 6 shows two kinds of heat flows at mass fractions from 0.36 to 0.40. In this range, the endothermic peak due to ice melting was not observed. From the mass fraction 0.36 to 0.39, hydrate dissociation peaks with similar shapes appeared at the temperature around 10°C. The depths of those peaks tend to slightly decrease with the increase in solution TBPOx mass fraction. At the mass fraction 0.40, the small endothermic peak and exothermic peak were observed at a temperature of around 5°C. After the appearance of those small peaks, the deep dissociation peak, similar to the mass fraction 0.36 to 0.39, emerged.

At the mass fraction range smaller than the hydrate composition, with increase of the mass fraction in solution, more hydrate crystals formed. Those crystals resulted in the larger peak due to hydrate dissociation. With the hydrate crystal formation, the amount of residual water in the aqueous solution decreased. Therefore, the ice melting peak tends to decrease as the mass fraction increased. This tendency was observed at the mass fraction range from 0.20 to 0.35 shown in Figure 4. When the mass fraction of the aqueous solution was equal to the hydrate composition, the largest amount of hydrate should be formed. After hydrate formation, no residual water existed in the test section. That means ice melting peak that emerged from the water may not appear at this mass fraction. Those results may lead to the deepest single peak of hydrate dissociation at a mass fraction of 0.35. At the mass fraction range larger than the hydrate composition, the amount of water required to form the ice exceeded the amount of the water in the aqueous solution. With increase in the mass fraction of the TBPOx in this aqueous solution, more water may be needed to form hydrate with all of the excess TBPOx present in solution. Due to the lack of water compared to the required stoichiometry, hydrate formation decreased. This would explain the decreasing peak depth of hydrate dissociation heats shown in Figure 6 at the mass fractions from 0.36 to 0.39.

As shown in Figure 6 at the mass fraction 0.40, the heat flow obtained by the heating rate of 2°C min^−1^ crossed the baseline. The two kinds of hydrate dissociation peaks were not separated at this heating rate. For a more accurate measurement, the dissociation measurement was performed with the heating rate of 0.5°C min^−1^ only at the mass fraction 0.40. In Figure 7, as well as the previous heat flow, two endothermic peaks of hydrate dissociation and one exothermic peak were obtained. Compared to the heat flow at 2°C min^−1^ heating rate, the exothermic peak at a temperature of around 5°C was obviously confirmed. The exothermic peak appeared just after the small endothermic peak. The endothermic peaks would emerge due to the hydrate formation. Although the heating rate was slower, two kinds of hydrate dissociation peaks were still not separated. The reaction of small endothermic, exothermic, and large endothermic processes continuously occurred. The small endothermic peak emerged because of the dissociation of the lower equilibrium temperature hydrate, and the large endothermic peak was due to the hydrate dissociated around 10°C. At the temperature around 5°C, the hydrate of the lower equilibrium temperature was dissociated into aqueous solution, and another type of hydrate of equilibrium temperature around 10°C also started to dissociate. On the endothermic dissociation process of the higher equilibrium temperature hydrate, the aqueous solution from the lower equilibrium temperature hydrate would be cooled. The dissociation of the higher equilibrium temperature hydrate and the recrystallization of the lower equilibrium temperature hydrate would occur simultaneously.

The dissociation heats, corresponding to the peaks in the DSC spectra near 10°C measured in this study, are shown in Table 3, where Δ*H*_d_ indicates the dissociation heats (enthalpies). The dissociation heats increased with the mass fraction at the mass fraction range below 0.35. In the present measurements, no ice melting peak appeared at a mass fraction of 0.30. The dissociation heat was obtained from the heat flow without the ice melting peak. At the mass fraction 0.35, the largest dissociation heat, 186.0 ± 0.5 kJ·kg^−1^, was obtained. At the mass fraction range from 0.35 to 0.37, the dissociation heats gradually decreased and then increased with the mass fraction at that range from 0.38 to 0.39. Over the mass fraction range 0.39, the dissociation heats decreased. The values of the hydrate dissociation heat resulted from the amount of hydrates formed in the test cell. The largest amount of hydrates was considered to be formed at the mass fraction 0.35.

**Table 3:** The dissociation heats of TBPOx hydrate from differential scanning calorimetry measurements.

***w*_**TBPOx**_^**a**^**	***x*_**TBPOx**_^**b**^**	**Δ*H*_**d**_ (kJ·kg^**−1**^)**	***U*^**c**^ (Δ*H*_**d**_) (kJ·kg^**−1**^)**
0.20	0.0074	77.8	±1.5
0.30	0.0126	145.1	±2.2
0.34	0.0151	180.9	±1.0
0.35	0.0157	186.0	±0.5
0.36	0.0164	178.3	±0.7
0.37	0.0171	179.4	±2.7
0.38	0.0180	181.8	±3.5
0.39	0.0186	181.7	±3.5
0.40	0.0194	176.0	±3.7

a *The standard uncertainty u (w_TBPOx_) is ± 5.0 × 10^−3^*.

b *The standard uncertainty u (w_TBPOx_) is ± 4.0 × 10^−3^. These values of mole fractions were obtained by converting the mass fractions*.

c *The expanded uncertainty U (ΔH_d_) (kJ·kg^−1^) was obtained by the triplicate measurements (k = 2)*.

**Figure 8:**
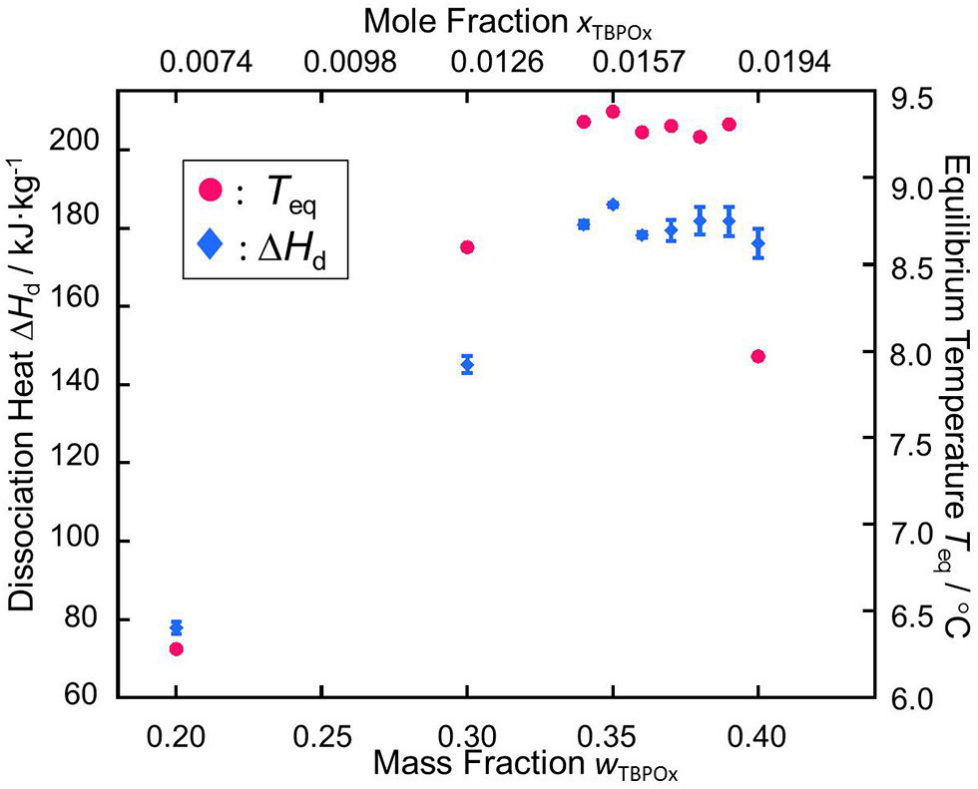
Relationships between the equilibrium temperature *T*_eq_ and the dissociation heat Δ*H*_d_ vs. the mass fraction of TBPOx aqueous solution *w*_TBPOx_ and the mole fraction of TBPOx aqueous solution *x*_TBPOx_: red •, the phase equilibrium temperature of TBPOx hydrate; blue ♦, the dissociation heat of TBPOx hydrate.

The measurement data of the phase equilibrium temperatures and the dissociation heats are summarized in Figure 8. In the phase equilibrium measurement, as well as the tendency of the dissociation heat, measured values of the thermophysical properties initially increased with the mass fraction. Over the mass fraction range from 0.35 to 0.39, the phase equilibrium temperature and dissociation heat tended to decrease. Those plots, respectively showed parabolic shapes, which was similarly confirmed in the previous studies (Yamauchi et al., 2017a,b; Arai et al., 2018, 2019; Shimada et al., 2018, 2019; Koyama et al., 2019).

The maximum points in the plots of phase equilibrium temperature and dissociation heat indicate that the congruent point of TBPOx semiclathrate hydrate would exist at the mass fraction range from 0.34 to 0.36.

### Evaluation as Thermal Energy Storage Medium

The measurements showed that the highest phase equilibrium temperature of TBPOx was 9.4°C and the largest dissociation heat was 186.0 ± 0.5 kJ·kg^−1^. To evaluate thermophysical property as a thermal energy storage medium, TBPOx could be compared with other PCMs, organic compounds, water, metallic salt hydrates, and other semiclathrate hydrates. Table 4 shows the latent heat of substances that could be utilized as cold energy.

**Table 4:** The equilibrium temperatures and dissociation heats of PCMs available as cold energy.

**Compound**	***T*_**eq**_ (^**°**^C)**	**Δ*H*_**d**_ (kJ·kg^**−1**^)**	**References**
Paraffin (C15–16)	8.0	153^a^	Lorsch et al., 1975; Mehling and Cabeza, 2007
LiClO_3_·3H_2_O	8.1	155^a^	Naumann and Emons, 1989; Veerakumar and Sreekumar, 2015
Water	0	333	Feistel and Wagner, 2006
Tetrabutylammonium bromide (TBAB) hydrate ^b^	12.7	193	Oyama et al., 2005; Kobori et al., 2015
Tetrabutylammonium chloride (TBAC) hydrate ^b^	15.0	201	Castellani et al., 2014
Tetrabutylammonium butylate (TBABu) hydrate	15.4	185	Yamauchi et al., 2017b
Tetrabutylammonium propionate (TBAP) hydrate	17.5	203	Yamauchi et al., 2017a
Tetrabutylphosphonium bromide (TBPB) hydrate ^b^	9.2	214	Suginaka et al., 2012
Tetrabutylphosphonium holoride (TBPC) hydrate ^b^	10.3	194	Sakamoto et al., 2011
Tetrabutylphosphonium propionate (TBPBu) hydrate	13.9	204	Shimada et al., 2019
Tetrabutylphosphonium propionate (TBPP) hydrate	15.6	190	Shimada et al., 2019
Tetrabutylphosphonium oxalate (TBPOx) hydrate	9.4	186	-

a *The value of the latent heat*.

b *The hydrate contains halide anions*.

The PCMs, paraffin (C15–16), and LiClO_3_·3H_2_O respectively represent organic compounds and metal salt hydrates, which have phase equilibrium temperature similar to that of TBPOx hydrate (Lorsch et al., 1975; Naumann and Emons, 1989; Mehling and Cabeza, 2007; Veerakumar and Sreekumar, 2015). In this temperature range, the latent heat of water is not available. The required energy 42.2 kJ·kg^−1^ was calculated by multiplying the specific heat of water 4.22 kJ kg^−1^·K^−1^ (IAPWS, 2008) with the temperature difference 10 K. As well as water, TBPOx hydrate also has the sensible heat. Adding the sensible heat to the dissociation heat, total energy density of TBPOx hydrate would be over 186.0 kJ·kg^−1^. This could help reduce the size and the mass of the practical device.

The energy density of TBPOx hydrate is approximately four times larger than that of water. Compared with other PCMs with close phase equilibrium temperature, TBPOx hydrate has the equivalent energy density. Furthermore, it has no flammability and corrosiveness unlike organic compounds and metal salt hydrates. While well-known semiclathrate hydrates, TBAB hydrate and tetrabutylammonium choloride (TBAC) hydrate, have large latent heat, they contain halide anions (Oyama et al., 2005; Sato et al., 2013). TBPOx hydrate includes carboxylic acid anions in the guest compounds. Compared to these compounds, TBPOx hydrate is environmentally friendly. With this safety and benign environmental aspect, TBPOx hydrate is suitable for the thermal energy storage media in air conditioning systems. Compared to hydrates incorporating carboxylic anions, TBPOx hydrate has a different equilibrium temperature from tetrabutylammonium butylate (TBABu) hydrate, tetrabutylammonium propionate (TBAP) hydrate, tetrabutylphosphonium butylate (TBPBu) hydrate, and tetrabutylphosphonium propionate (TBPP) hydrate. Although the value of the latent heat is slightly lower than them, TBPOx could extend the temperature range for the application.

## Conclusion

In this study, thermophysical properties of TBPOx hydrate were measured. The highest phase equilibrium temperature was 9.4°C at the mass fraction 0.35 in aqueous solution. The largest dissociation heat was 186.0 ± 0.5 kJ·kg^−1^ at the mass fraction 0.35. Incorporating carboxylic acid anions as parts of the guest compounds, it has a more environmentally friendly composition than halide-based alternatives. As a PCM, the energy density of TBPOx hydrate is equal to or greater than other substances having close phase equilibrium temperature. Moreover, having no flammability and corrosiveness unlike organic compounds and salt metal hydrates, it is useful in the aspect of safety and sustainability. TBPOx hydrate would be suitable as a thermal energy storage media for the use of air conditioning system.

## Data Availability Statement

All datasets presented in this study are included in the article/supplementary files.

## Author Contributions

The experiment design and manuscript preparation were done by TM with RO. NK and AH assisted with the experiments in DSC measurement. SA and RO assisted in experiment design, data analysis, and the preparation of manuscript. All authors contributed to the article and approved the submitted version.

## Conflict of Interest

The authors declare that the research was conducted in the absence of any commercial or financial relationships that could be construed as a potential conflict of interest.
